# Mississippi church leaders' perceptions of challenges and barriers to the use of consumer wearables among community members

**DOI:** 10.3934/publichealth.2023052

**Published:** 2023-10-07

**Authors:** Monique White, Candis Pizzetta, Edith Davidson, Andre Hines, Mario Azevedo, Fidelis Ikem, Lena M. Jones, Shelia Malone, Girmay Berhie

**Affiliations:** 1 Public Health, Informatics, and Technology, College of Health Sciences, Jackson State University, Jackson, MS, USA; 2 Department of English, Foreign Languages, and Speech Communication, College of Liberal Arts, Jackson State University, Jackson, MS, USA; 3 Department of Business Administration, College of Business Administration, Jackson State University, Jackson, MS, USA; 4 Department of Public Policy & Administration, College of Liberal Arts, Jackson State University, Jackson, MS, USA; 5 Department of History and Philosophy, College of Liberal Arts, Jackson State University, Jackson, MS, USA; 6 Department of Epidemiology and Biostatistics, College of Health Sciences, Jackson State University, Jackson, MS, USA

**Keywords:** consumer wearables, health disparities, African Americans, technology, church leaders', perceptions, Mississippi

## Abstract

**Background:**

Wearables have begun to play a transformative role in health management and disease prevention.

**Objective:**

This study examined the use of wearable devices in African American communities in Mississippi, USA, through the lens of church leaders.

**Methods:**

We conducted focus groups with church leaders to record their perceptions about the use of wearables of their community members. We conducted six focus groups with a total of 89 church leaders from across the state of Mississippi. The focus groups were designed to contextualize and explain the socio-cognitive processes that provided an understanding of wearable device adoption practices among community members. Participants were male and female church leaders who were recruited from the three Mississippi Districts. The church leaders' perceptions of barriers and challenges to the adoption of consumer wearables in their communities were thoroughly analyzed using thematic analysis.

**Results:**

There was great apprehension on the part of community members about the security of the information they entered into the wearable devices and about how that information could be used by other parties. Many community members who understood the value of proactive health behaviors could not afford the high cost of purchasing wearable devices, while others displayed a low level of comfort with technology, believing that wearable use was for younger people.

**Conclusion:**

More expansive adoption of wearable devices in Mississippi will depend on the ability of the public health professionals, policy-makers and manufacturers to address the barriers that were identified by this study, thereby enabling the community to have full access to the potential benefits of these technologies.

## 1. Introduction

In the United States of America (USA), wearable technologies, specifically defined as compact electronic devices that can be conveniently attached to the body or clothing [1], are surging in popularity. Wearables have begun to play a transformative role in health management and disease prevention. Health wearable devices are recommended for a wide range of health domains, such as predicting infections, tracking fertility cycles, monitoring heat-related illnesses, issuing alerts about potential drug effects, and even aiding in psychological interventions [2]. They present significant advantages for health research. This study focuses on gaining an understanding of the use of wearable devices by African American communities in Mississippi, USA.

Wearables are effective tools to help improve cardiovascular health through enhanced self-monitoring. Evidence shows that when people utilize wearables, they may participate in more physical activities. The increase in wearable usage is attributed to the belief that wearable devices have the potential to positively impact health behaviors and facilitate innovative health research. Wearable technologies represent a major step towards bridging the gap between scientific research and everyday health management [3]–[5].

Achieving widespread utilization of wearable technology poses a significant challenge for health professionals, particularly in underserved communities at risk for developing chronic diseases, like those within the African American community [6]. A report by the American Heart Association (AHA) indicated that people who need wearable health devices the most use them the least [7]. The factors influencing successful implementation of wearables and the barriers impeding their adoption are not fully understood [8],[9]. Hence, one of the motivating factors for initiating this study in Mississippi, a state where African Americans experience the highest prevalence of chronic disease, particularly cardiovascular disease (CVD).

African American communities have a higher prevalence of cardiovascular disease and other health disparities, compared to the general population. This is primarily because of the lack of culturally sensitive interventions that they can fully endorse and effectively promote. For most African American communities, culturally sensitive interventions are initiated in the church [10],[11]. African American church leaders in Mississippi have traditionally held a crucial role of influence and motivation regarding health behavior of their community members [12]. Church members view their church leader as a trustworthy community resource and a major influence on their health behavior [13],[14]. We sought to secure the perceptions of church leaders for this study because African American church leaders have traditionally embraced a significant role to organize and promote community participation in health matters. Also, their interaction with their congregations makes them knowledgeable enough about their community to objectively report on health issues, risk behaviors and the consensus of their community regarding strategies to address risk factors for prematurely developing chronic diseases.

According to the Mississippi State Health Department (MSDH), their Health Equity Division is committed to addressing health equity in the processes, policies and procedures of all MSDH programs. In the struggle to achieve equity, they stress that it is important to work with external partners, like church leaders, to identify health disparities, challenges and barriers, seek innovative solutions to address them and view and develop initiatives with health equity as a factor to help guide policy development [15]. Therefore, it is important to identify effective public health strategies that bridge the health gap.

Specifically, for AA populations, the context of health is culturally defined and will require culturally aligned interventions to holistically address the health status of this population. Health promotion programs operated through churches are increasingly being touted as effective in promoting healthy living among AAs [11]. Religious institutions, particularly churches, have traditionally held an influential position in shaping both individual and community behaviors. This extends notably to the realm of health, where churches possess substantial potential to ameliorate health disparities through various initiatives encompassing health promotion, education and intervention. The church serves as an important platform for communicating and reinforcing health-related messages within the community, bolstered by its spiritual and cultural significance. The participation of many African Americans in health interventions underscores the intersection of faith and wellness within these communities. These interventions often weave together spiritual and cultural elements, forming a unique approach to health promotion that resonates deeply with the participants. Such interventions have proven to be efficacious in facilitating positive alterations in health behaviors [16]. It is the fusion of health promotion strategies with culturally resonant spiritual elements that represent the type of supportive environment that encourages behavior change. In a qualitative study conducted by Baruth et al. [17], the influence of faith leaders on the health of their congregations was examined. Of the 24 participants, the majority reported that health-related activities were a common feature within their churches. This observation underscores the prominent role that religious institutions occupy within these communities, particularly in matters concerning health and wellness. By raising awareness about key health issues and promoting proactive strategies to address them, church leaders have the ability to significantly impact the overall health status of their community. In recognizing this potential, health interventions can be designed to further leverage the influence of church leaders to promote health, ultimately improving health outcomes within the community.

According to the Centers for Disease Control and Prevention (CDC), African American communities are faced with limited access to health promotion resources, while they bear a disproportionate burden of preventable diseases [18]. The African American church leaders provide the types of multilevel culturally aligned strategies that are required to address these challenges successfully. Recognizing the influential role that religious institutions and their leaders play in shaping health behaviors, we specifically sought the collaboration of church leaders in districts across the state of Mississippi for this crucial research study. Our objective was to understand the barriers hindering African Americans in Mississippi from using wearable devices for health monitoring purposes. Our focus on church leaders in this project was intentional because of their traditionally deep understanding of their communities and their ability and willingness to influence the adoption of health-promoting behaviors. We are interested in exploring their perceptions of the barriers that might be preventing the use of wearables. The goal of this research was to gain insights into the views of these church leaders concerning the obstacles, challenges and barriers faced by African Americans in acquiring and utilizing wearable devices. We aim to understand these barriers and challenges from both a socio-cultural and technological perspective, looking into factors such as accessibility, cultural appropriateness, technological literacy and potential privacy concerns. With this understanding, we hope to gain insight into potential strategies that would facilitate the increased adoption of wearable devices for health monitoring, ultimately supporting improved health outcomes for individuals and African American communities in Mississippi. We hope that our findings can contribute to interventions that are culturally appropriate, effective and sustainable, leveraging technology to enhance the health and wellbeing of these communities.

This study was devised to take a closer look at the use of wearable devices in African American communities in Mississippi, USA, because it has been reported that wearable devices grant individuals the capacity to actively monitor their physical activity while recording routine workouts and tracking health risk practices; wearables collect essential health data that provide personalized and actionable insights. This data collection facilitates an understanding of community members' health and wellness journey. If community members practice tracking personal progress in real-time, they would be empowered to make necessary adjustments to their behaviors and practices, thereby paving the way for improved health status.

## 2. Methods and materials

### 2.1. Study design

A qualitative research design was deemed appropriate for this study. Most of the 89 church leaders who provided usable responses (85.4%) were in the age range 35 years old and above. We conducted focus groups with our church leaders to record their perceptions about the use of wearables of their community members to address the problem of this study.

### 2.2. Theoretical model

This study was initiated to gauge the perceptions of church leaders to better understand the challenges and barriers faced by their community members regarding the use of wearables. The study was guided by basic tenets of two theoretical models that guided the examination of perceptions of church leaders regarding their community members' beliefs and practices relating to the use of wearable devices among their communities for the purpose of addressing risk factors for the development of diseases:

1. Elements of the social-ecological model (SEM) served as a solid backdrop to understand from the church leaders the personal and environmental factors that prevented community members from utilizing wearable intervention strategies to reduce health disparities. Individual health and health behaviors and strategies result from multiple sources of family, work, community and political influences [16],[19],[20]. SEM provides a background and framework for exploring how decisions regarding health are made. According to this model, when church leaders preach against unhealthy behaviors and provide health education, preaching along with supporting health promoting strategies, they are able to alert their communities about healthy living and can influence beliefs, attitudes and behavior change at multiple levels. This enables the church leaders to develop an understanding of their community members health behavior practices.

2. This study also explored the challenges and barriers associated with the use of wearable devices through the lens of the transtheoretical model of behavior change (TTM). Understanding the barriers and challenges to the use of wearable devices is equally important in the struggle to eliminate health disparities. The TTM proposes that change is a process that unfolds over time and progresses through a series of stages [21]–[23]. In the early stage, people do not plan to change their behavior in the near term because they are either uninformed or underinformed about the consequences of their behavior. Some people might not be aware of the existence of wearable devices or realize what health benefits wearable use entails. The costs and beneﬁts of change potentially produce uncertainty, and some people remain in an uncertain phase for extended periods of time. For instance, wearable use could be perceived as beneficial but the price and lack of technology skills could stop some people from adopting it.

### 2.3. Sample

We conducted 6 focus groups with a total of 89 church leaders from across the State of Mississippi. The focus groups were designed to contextualize and explain the socio-cognitive processes that provided an understanding of wearable device adoption practices among community members. Participants were male and female church leaders who were recruited from the three Mississippi Districts: the Northern District, the Central District and the Southern District. The Northern District included Alcorn, Bolivar, Calhoun, Clay, Coahoma and Grenada counties. The Central District included Choctaw, Claiborne, Clarke, Copiah, Hinds, Madison and Rankin counties. The Southern District included Adams, Amite, Covington, Forrest, George, Harrington, Jackson, Lamar and Pike counties. Focus groups were held face-to-face as well as virtually, using ZOOM. Table 1 below describes the demographics of the church leaders who participated in the study.

### 2.4. Instrument

During the focus groups, the Mississippi church leaders were asked to provide their perceptions on potential challenges and barriers related to wearable of their community members. Specifically, they were asked to address the following seven socio-cognitive concerns that could influence their practices:

a. Safety: The degree to which wearables posed any risk, harm and injury.

b. Security: The safekeeping of personal data collected by the devices.

c. Privacy: The assurance that user data would not be exposed or shared without explicit consent

d. Equity: The equal opportunity for community members to access health services.

e. Trust: The confidence that the devices deliver reliable information.

f. Instrument or device accuracy: The reliability of information obtained from the wearable device

g. Other barriers: Any additional challenges not covered in the above categories.

Data was collected in phases and from different sources. The primary variable of interest was the Mississippi church leaders' perceptions of barriers to the use of wearable devices among community members. The focus group interviews served to address the question about the perceptions of the church leaders about the challenges and barriers they faced regarding wearable device use. Six different focus groups were held with church leaders from different districts around Mississippi at pre-scheduled times. The church leaders participating in the focus represented church leaders represented the different districts around Mississippi. Focus groups for each district were held at pre-scheduled times. The responses of the church leaders were recorded, transcribed and anonymized for further analysis. Data were then summarized using thematic analysis, thereby detailing the prevailing themes.

The interview transcriptions were reviewed by one of the investigators to verify their accuracy. They were coded by two other investigators. Differences between coders were resolved through discussions. Analysis was conducted using thematic analysis [24],[25].

### 2.5. Data analysis

The data analysis for this study was conducted on responses from all church leaders who participated in the focus groups. The responses to the demographic section and the responses to the target area of interest were reported quantitatively using frequencies and percentages. For the demographic section, we examined the varied characteristics of the church leaders, including their age, race, education level, denomination, and role in the church among others. This data was reported quantitatively using frequencies and percentages, providing an overview of the demographic profile of our participants.

As for the target area of interest, the church leaders' perceptions of barriers and challenges to the adoption of consumer wearables in their communities were thoroughly analyzed. This encompassed their views on issues related to safety, security, privacy, equity, trust, device accuracy and other challenges related to wearable technologies. Responses to these themes were quantitatively reported, again using frequencies and percentages to identify dominant views and trends.

The results of the analyses are presented in Table 1, “Demographic Characteristics of Church Leaders” and in Table 2, “Church Leaders' Perceptions of Barriers to Wearable Use,” both of which are included below in Results.

## 3. Results

The church leaders provided responses that identified ten areas that they perceived to be challenges and barriers. Tables 1 and 2 below provide the results of the analyses that were computed on the responses provided by the church leaders who participated in the focus groups. Table 1 is a presentation of the demographic characteristics of the church leaders who participated in the study. As seen in Table 1, the aged of the church leaders ranged from 18–65 years old and older. The largest group of church leaders (33.7%) were in the 45–54 age range, followed by 25.4% in the 55–64 age group and 18.4% of them in the 35–44 age group. About 2.2% of them were 18–24 years old, 12. 4% were 25–34 years old and 7.9% of them were 65 years of age or older. The group of church leaders comprised 60.7% male participants and were 98.9% African American. Over 60.0% of them were married and 42.7% of them had a bachelor's degree.

Nine denominations were represented in this study with the Baptist group having the largest number of participants (67.4%). About 38.2% of the church leaders represented churches that comprised 101–200 members and 33.7% of them had a congregation of 100 members or less. The church leaders occupied a variety of roles within their respective churches with 47.2% of them operating as pastors, 15.7% as youth leaders and 11.2% as deacons.

**Table 1.** Demographic characteristics of church leaders.

**Variable**		**Number**	**Percentage**
**Age**			
	18–24	2	2.2
	25–34	11	12.4
	35–44	16	18.0
	45–54	30	33.7
	55–64	23	25.8
	65 or older	7	7.9
**Gender**			
	Female	35	39.3
	Male	54	60.7
**Race**			
	Black/African American	88	98.9
	Hispanic of any race	1	1.1
**Marital Status**			
	Divorced	5	5.6
	Married	54	60.7
	Separated	2	2.2
	Single	22	24.7
	Widowed	6	6.7
**Education**			
	Associate degree	8	9.0
	Bachelor's degree	38	42.7
	Graduate or professional degree: ________	8	9.0
	High school graduate or GED	4	4.5
	Prefer not to answer	3	3.4
	Some college	25	28.1
	Trade or vocational degree	3	3.4
**Denomination of Church**			
	AME	1	1.1
	Baptist	60	67.4
	Catholic	1	1.1
	Church of God in Christ-Pentecostal	5	5.5
	Methodist	1	1.1
	Missionary Baptist	2	2.2
	Non-denomination	13	14.6
	Pentecostal	3	3.4
	United Methodist	3	3.3
**Congregation Size**			
	0–100 members	30	33.7
	101–200 members	34	38.2
	201–300 members	16	18.0
	301–500 members	8	9.0
	More than 500 members	1	1.1
**Role with Church**			
	Missing	1	1.1
	Assistant pastor	3	3.3
	Associate minister	1	1.1
	Associate pastor	1	1.1
	Choir director/WIA leader	1	1.1
	Deacon	10	11.2
	Elder	3	3.4
	Executive secretary	1	1.1
	First lady	1	1.1
	Leader	1	1.1
	Minister	3	3.4
	Na	1	1.1
	Pastor	42	47.2
	Pastor council	1	1.1
	Pastors council leader	1	1.1
	Praise team	1	1.1
	Secretary	2	2.2
	Yard person	1	1.1
	Youth leader	14	15.7

Table 2 presents the ranking of the top ten barriers observed by the Mississippi church leaders ranging from the highest to the lowest. They are the following:

1. More than half of the church leaders have exposure concerns.

2. Half of Church leaders do not trust the accuracy of the information from the devices.

3. Some church leaders think affordability is a barrier to wearables.

4. Some church leaders are concerned about unauthorized tracking.

5. Most of church leaders think that trust and privacy concerns are barriers to wearables.

6. Some of the church leaders said that they are uncomfortable with the GPS feature.

7. Some of the church leaders believe that African Americans are not receptive to consumer wearables.

8. Church leaders think that the devices are distractive and may pose health hazards.

9. Church leaders feel that consumer wearable use is a generational thing and that they are used as accessories by younger generations.

10. Some of the church members feel that African Americans' lack of education about the device is a factor of “resistance to use”.

**Table 2.** Church leaders' perceptions of barriers to wearable use.

**Rank**	**Church Leader's Perceptions**	**Percent (%)**
1	Church leaders have exposure concerns.	55
2	Church leaders do not trust the accuracy of the information from the devices.	50
3	Church leaders believe that affordability would pose a problem regarding health equity.	44
4	Church leaders are concerned about unauthorized tracking.	40
5	Church leaders think that trust and privacy concerns is a barrier to wearables.	25
6	Church leaders said that they are uncomfortable with the GPS feature.	22
7	Church leaders feel that the African Americans are not receptive to consumer wearables.	21
8	Church leaders think that the devices are distractive and may pose health hazards.	20
9	Church leaders feel that consumer wearable use is a generational thing and that they are used as access younger generations.	15
10	Church leaders feel that African Americans lack of education about the device is factor of “resistance to use”.	14

The key findings projected by the church leaders are the following:

**1.** The interviews showed that the church leaders perceived that there was great apprehension on the part of their community members about the security of information they entered in the wearable devices and great concern about how that information could be used by other parties. The church leaders believed that these concerns and other concerns relating to tracking of their health information had an impact on community members' decision about using of wearable devices.

**2.** The church leaders also believed that the lack of education about the benefits and value of the wearable devices created anxiety in community members about the use of wearable devices. After using the devices, as the second set of interviews showed, seniors were more concerned with the output complexity of the wearable device.

**3.** The church leaders also indicated that the majority of their community members could not afford the high cost of purchasing wearable devices.

**4.** Another factor observed by the church leaders was that their community members displayed a low level of comfort with technology believing that wearable use was for younger people.

## 4. Discussion

This research sought to understand the challenges and barriers of wearable devices of community members from the perspectives of church leaders. We sought to use the perceptions of church leaders for this study because African American communities are faced with limited access to health promotion resources, and bear a disproportionate burden of preventable diseases [18]. As a result, the African American church leaders have traditionally accepted the role of encouraging and providing the kind of multilevel culturally aligned strategies that are required to motivate their communities to successfully address these health challenges. Therefore, these church leaders have a profound understanding of the factors that motivate or prevent their community members' decisions regarding wearable device use.

We used a qualitative research design, using focus groups, to engage church leaders from the districts in the state Mississippi, USA. Six focus discussions were held in-person as well as virtually, via ZOOM. The interviews revealed that the church leaders perceived that there was great apprehension on the part of their community members about the security of information they entered in the wearable devices and great concern about how that information could be used by other parties. The church leaders believed that these concerns and other concerns relating to tracking of their health information had an impact on community members' decision about using of wearable devices. The church leaders also believed that the lack of education about the benefits and value of the wearable devices created anxiety in community members about the use of wearable devices. Subjective well-being plays a major role in seniors' adoption of wearable devices [26]. The participants in their study believed that using wearable devices can improve their performance compared to their peers. The church leaders in our study indicated that the many of their community members who understood the value of proactive health behaviors could not afford the high cost of purchasing wearable devices, while some others displayed a low level of comfort with technology, believing that wearable use was for younger people. Mississippi church leaders perceive that some community members believe that wearable device use is more prevalent in younger generations who often use these devices as accessories rather than health tools. They also believe that a potential knowledge gap exists among African Americans and that lack of education about the devices could contribute to reluctance or “resistance to use.” Colvonen et al. reported about the Black Lives Matter movement's plea to remove systemic bias by challenging the healthcare community to work ensuring that the use of wearable devices do not reinforce existing disparities in care and access with the increasing use of wearable devices in research and clinical practice [27].

Another barrier for community members, as indicated by the Mississippi church leaders, was concern regarding privacy, notably the potential exposure of sensitive personal information. The state of Mississippi, a state with one of the highest prevalence of obesity and CVD, actively encourages community members to use wearable devices to improve their health status. Wearable devices can aid in monitoring nutritional intake, physical activity and various physiological parameters, empowering individual community members and potentially helping to decrease obesity [28]. As Rossi disclosed, with the increasing popularity of wearable devices, individuals can sometimes provide their personal, sensitive information in order to position themselves to gain an advantage from the analysis of their health [29]. This finding is consistent with the perceptions of the church leaders' that some community members fear that engaging in this practice could lead to legal questions and legal problems regarding privacy and product liability.

The Mississippi church leaders also indicated that they observed that there was a general lack of trust in the accuracy of the information from the devices use by their community members. With the increasing application of wearable technologies used for clinical research and healthcare, accuracy is important because measurement errors can impact conclusions and impact healthcare decision-making, a finding that is supported by research conducted by Bent et al. [30]. The results of this study indicate that church leaders perceive that some community members may not be confident that wearable devices will assess and report their information accurately.

Church leaders also think that affordability is a barrier to the wide adoption of wearables and that this barrier will impact health equity. Even though wearable technologies are increasingly popular, their use continues to be by older adults who can benefit the most from utilizing wearable devices [31]. While companies advertise widely for the use of wearable devices as a healthcare support device, they will only be effective if individuals wear these devices as recommended [32]. The church leaders suggested that cost could deter a significant proportion of the community from using these devices, thereby limiting their impact.

Mississippi church leaders were concerned about unauthorized tracking, which presents another barrier in regards to privacy/confidentiality of collected data from wearables usage [33]. The church leaders think that trust and privacy concerns are barriers to wearables, and this is supported by concerns expressed at the federal level. In September 2021, the Federal Trade Commission issued the following policy statement that health apps and connected devices that collect or use consumers' health information must comply with the Health Breach Notification Rule, which requires that they notify consumers and others when their health data is breached. In a policy statement adopted during an open meeting, the commission noted that health apps, which can track everything from glucose levels for those with diabetes to heart health to fertility to sleep, increasingly collect sensitive and personal data from consumers These apps have a responsibility to ensure they secure the data they collect, which includes preventing unauthorized access to such information [34]. Concerns regarding trust and privacy as related to data security were reflected in the responses of the church leaders and appear to reflect an awareness of potential data breaches and misuse. Mississippi church leaders were also uncomfortable with the GPS feature of wearable devices, which allows tracking of an individual, and they also believe that African Americans are not receptive to consumer wearables, in part because of this feature.

The church leaders' role to monitor and manage their community members' health behaviors is an important one in view of some of the findings that have been reported in the literature. Colvonen et al. and Reddy et.al. reported reduced accuracy of wearable devices in people with darker skin tones due to limitations in the design of the wearables [27],[35]. Many health wearables are designed and tested primarily on lighter skin tones. The resultant discrepancies in data collection for darker skin tones can lead to inaccuracies, especially for functionalities like heart rate monitoring that rely on optical sensors. Skin pigmentation can influence the absorption and reflection of light, potentially affecting the accuracy of these readings (Bent et al., 2020). It is important that steps be taken to reduce existing structural health disparities of people with darker skin tones, particularly African Americans [36]. It has been reported that some devices lack accuracy in people with dark skin tones, a situation that denotes a gap in access that has to be addressed by manufacturers.

Mississippi church leaders also think that some wearable devices are distractive and may pose health hazards. This belief is consistent with previous findings by Smuck who proposed that deficits in knowledge have resulted in concerns that “wearables could pose health risks by paradoxically reducing healthy behavior rather than promoting it” [6].

## 5. Strengths

This study contributes to the current literature on wearable devices highlighting the factors that may inhibit the choice and intention of African Americans in Mississippi to use wearable devices. The main contribution of this study is the presentation of information that can be used by health agencies, such as the Mississippi State Department of Health, as they plan strategies to partner with groups of community advocates to actively campaign to reduce risk factors for development of disease by advocating the independent, proactive use of wearable devices to monitor health status. Older community members are usually the group that is most affected by the economic and healthcare consequences of disease, the significantly higher prevalence, and death rates compared to other demographic groups. The results support previous research that illustrate that wearable devices have the potential to help in monitoring health and activity, if practitioners make the effort to expand their use.

## 6. Limitations

There are limitations to the study that should be acknowledged. The results of this study cannot be used to generalize behaviors of all residents of the state of Mississippi or of all African Americans. Our focus group interviews presented the critical roles of selected factors in the willingness and ability of African American community members to use wearable devices through the lens of the church leaders and not the community members themselves. Future research can take a broader perspective and include community members' perceptions that can then be compared to the church leaders' perceptions to extend the model we developed.

## 7. Conclusions

In this study, we focused on understanding elements that impact African American community members' intention to use wearable devices. Previous literature has outlined numerous benefits of using wearable devices, but the widespread use among African Americans has not been substantiated. So, it is critical for community members and healthcare professionals to understand the factors that can discourage community members from using wearable devices. In general, wearables are valuable devices that can generate data and make that information available for average community members to gather information that can assist them in managing their health status. With wearable devices like the Apple Watch, Fitbit and Samsung, individuals can track activity, sleep and other health-related outcomes. Wearables provide individuals with health-related information, such as problem heart rate alerts, a personal electrocardiogram (ECG) monitor for detecting arrhythmia, and pulse pressure. These and other physiological data tracking support healthy living and provide immediate notifications to wearable users at high-risk for health concerns. Wearable devices have great benefits for individual management of health status as well as clinical practice [27],[37].

This research focused on the barriers perceived by the Mississippi church leaders that can limit the use of wearable devices among community members. There are many barriers presented that militated against the utilization of wearable devices based on the observation of the church leaders. The most significant factors are related to exposure concerns, the accuracy of the information from devices, affordability of the devices and unauthorized tracking of data and information. More expansive adoption of wearable devices in Mississippi will depend on the ability of the public health professionals, policy-makers and manufacturers to address the barriers that were identified by this study, thereby enabling the community to have full access to the potential benefits of these technologies. These results expand the literature on wearable device use by providing a reflection of decisions and practices in African American communities in Mississippi, communities with the highest prevalence of cardiovascular diseases in the USA, struggling to reduce health disparities.

## Use of AI tools declaration

The authors declare they have not used Artificial Intelligence (AI) tools in the creation of this article.
